# Skin-Whitening and Anti-Wrinkle Effects of Bioactive Compounds Isolated from Peanut Shell Using Ultrasound-Assisted Extraction

**DOI:** 10.3390/molecules26051231

**Published:** 2021-02-25

**Authors:** Da Hye Gam, Ji Woo Hong, Jun Hee Kim, Jin Woo Kim

**Affiliations:** 1 Department of Food Science, Sunmoon University, Natural Science 118, 70 Sunmoon-ro 221, Tangjeong-myeon, Asan-si, Chungnam 336-708, Korea; ank7895@naver.com (D.H.G.); hgw130@naver.com (J.W.H.); jun981014@naver.com (J.H.K.); 2 FlexPro Biotechnology, Natural Science 128, 70 Sunmoon-ro 221, Tangjeong-myeon, Asan-si, Chungnam 336-708, Korea; 3 Center for Next-Generation Semiconductor Technology, Sun Moon University, 70 Sunmoon-ro 221, Tangjeong-myeon, Asan-si, Chungnam 336-708, Korea

**Keywords:** peanut shell, optimization, skin-whitening, anti-wrinkle, antioxidant, tyrosinase, collagenase, human tyrosinase related protein-1 (TRP-1), matrix metalloproteinase (MMP)

## Abstract

Response surface methodology was employed to optimize the ultrasound-assisted extraction (UAE) conditions for simultaneous optimization of dependent variables, including DPPH radical scavenging activity (RSA), tyrosinase activity inhibition (TAI), and collagenase activity inhibition (CAI) of peanut shell extracts. The effects of the main variables including extraction time (5.0~55.0 min, X_1_), extraction temperature (26.0~94.0 °C, X_2_), and ethanol concentration (0.0%~99.5%, X_3_) were optimized. Based on experimental values from each condition, quadratic regression models were derived for the prediction of optimum conditions. The coefficient of determination (R^2^) of the independent variable was in the range of 0.89~0.96, which demonstrates that the regression model is suitable for the prediction. In predicting optimal UAE conditions based on the superimposing method, extraction time of 31.2 min, extraction temperature of 36.6 °C, and ethanol concentration of 93.2% were identified. Under these conditions, RSA of 74.9%, TAI of 50.6%, and CAI of 86.8% were predicted, showing good agreement with the experimental values. A reverse transcription polymerase chain reaction showed that peanut shell extract decreased mRNA levels of tyrosinase-related protein-1 and matrix metalloproteinase-3 genes in B16-F0 cell. Therefore, we identified the skin-whitening and anti-wrinkle effects of peanut shell extracts at protein as well as gene expression levels, and the results show that peanut shell is an effective cosmetic material for skin-whitening and anti-wrinkle effects. Based on this study, peanut shell, which was considered a byproduct, can be used for the development of healthy foods, medicines, and cosmetics.

## 1. Introduction

Melanin is a brown-or black-colored polymer pigment that is synthesized from the melanosomes of melanocytes in the epidermis. Its main function is to block ultraviolet (UV) rays in order to protect the skin. Alternatively, its excessive production can cause pigment darkening, such as melasma, moles, and age spots [1,2,3]. Tyrosinase is a major enzyme that catalyzes autooxidation and polymerization reactions, through which tyrosine is converted into dopaquinone via dihydroxyphenylalanine and produces melanin through dopachrome during melanin biosynthesis [4]. Thus, it is widely used to reduce or attenuate melanin production through the inhibition of tyrosinase activity in order to enhance the whitening effect of cosmetics [5]. Rapid industrialization and increased use of chlorofluorocarbons severely damaged the earth’s protective ozone layer, thus resulting in greater amount of UV reaching the ground and exposing the skin. This increase in UV radiation consequently induces active generation of reactive oxygen species (ROS) in the human body, such as superoxide anions, hydrogen peroxides, and hydroxyl radicals. Such species have promoted the continuous oxidation of tyrosine, resulting in an increased production of melanin. In this regard, studies are actively conducted on the inhibition of tyrosinase activity as well as the removal of ROS in order to develop skin-whitening agents [6]. Collagen is a major extracellular matrix that comprises 90% of the dermis. Collagen protects and gives elasticity to the skin and is involved in the mechanical rigidity of the skin, resistance, and binding of connective tissues, and proliferation and differentiation of cells [7]. Proteins that make up extracellular matrix, such as collagen, are decomposed by collagenase, such as matrix metalloproteinase (MMP), causing wrinkles, decreased elasticity, and sagging of the skin [8]. Various types of MMPs that are expressed by ROS hydrolyze the collagen chain, skin connective tissue, and generate its abnormal crosslinking to increase collagen decomposition and accelerate the formation of wrinkles [9]. For this reason, inhibition of melanin production and collagen decomposition through reduction of ROS generation has been the main focus for skin whitening and wrinkle prevention [10]. Arbutin, kojic acid, and linolenic acid, as whitening cosmetics and retinol, gallate, and adenosine, as anti-wrinkle cosmetics, have been widely used in recent years. However, the use of these materials is limited, given their instability in the presence of light and heat as well as to adverse reactions, including skin irritation and contact dermatitis [11]. With growing interest in natural antioxidants for overcoming the shortcomings of conventional whitening and anti-wrinkle ingredients, plant-derived extracts have been actively used to develop bioactive compounds for skin-friendly and safe whitening and anti-wrinkle cosmetics [12,13].

There are various extraction methods that are currently used for the extraction of bioactive compounds from plants. However, the extraction of bioactive compounds from natural sources, especially plants, has mainly been conducted through solvent, hot-water, and Soxhlet extraction methods, which have shown various drawbacks, including low extraction efficiency, ingredient decomposition, low stability, and high cost of operation [14]. Thus, extraction methods have recently been tested, including ultrasound-assisted, microwave-assisted, and supercritical extraction [15]. In particular, ultrasound is a soundwave with a frequency of approximately 20 kHz or more, which results in compression, cavitation, and rarefaction of liquid, thereby maximizing the molecular motion in a short time to obtain high extraction efficiency [16]. Furthermore, ultrasound is advantageous, in that its short extraction time minimizes the decomposition of bioactive compounds and it is evaluated as an effective method for extracting natural ingredients with antioxidant, whitening, and anti-wrinkling properties from many plants and herbs [17]. Extraction condition optimization is essential to increase the efficiency of ultrasound-assisted extraction (UAE), and the optimization process can be performed either by experimental or statistical methods. The traditional one-factor-at-a-time method, with all variables remaining constant and changing only one factor at a time, has limitations in determining the interactive effects if it is a multivariate experiment. On the other hand, RSM provides statistical information on the correlation between variables in multivariate experiments, along with effective experiments using minimal number of samples, as well as important mathematical and statistical techniques for evaluating the effectiveness and suitability of the regression model. For statistically-based optimization, various RSM designs, such as full factorial design, Box-Behnken design, and central composite design (CCD), have been widely used. Among them, CCD is very efficient and thus provides much information on experiment variable effects and overall experimental error, with a minimum number of required runs [18]. Therefore, in many existing studies, CCD has been widely used to develop, improve, and optimize the process conditions for extracting various antioxidants and other metabolites from natural products.

Peanut (*Arachis hypogaea*) is an annual plant belonging to the legume family. It is grown in more than 50 countries around the world, including South Korea, India, China, and the United States [19]. Peanuts are rich sources of protein (25%), lipids (47%), and carbohydrates (16%), as well as minerals, vitamins, niacin, unsaturated fatty acids, and oleic acids [20]. They are consumed as either unprocessed or processed products, including nut, butter, and cooking oil. It is estimated that the world’s annual peanut production amounts to 4.1 million tons in total and that the peanut‘s shell accounts for 35%~40% of total weight of the peanut [21]. It is estimated that more than 1.5 million tons of peanut shells are discarded annually as byproducts. However, given that only a portion of peanut shells are used as animal feed and that most of them are incinerated or landfilled, causing disposal cost and environmental problems, it is necessary to produce high value-added materials using peanut shells to overcome the problem of byproducts [22]. Previous studies on antioxidants have shown that anti-inflammatory and anti-obesity activities of peanut skin extracts have been reported [23,24]. However, so far, there is no research on the production of functional cosmetic materials for improving whitening and anti-wrinkling effects using bioactive compounds from peanut shells. Therefore, this study extracted bioactive compounds from a peanut shell by employing the ultrasound-assisted extraction (UAE) to confirm their antioxidant, whitening, and anti-wrinkle effects and further presented an optimal UAE condition using the response surface method (RSM) and increased the functionality of extracts in order to confirm the possibility of its use as food, cosmetics, and medical ingredients.

## 2. Results and Discussion

### 2.1. Fitting the RSM Models

In this work, extraction temperature, extraction time, and ethanol concentration were selected as the main variables of CCD using the preliminary one-factor-at-a-time experiment to determine the significant variables affecting UAE (Table 1).

Then, 17 experimental runs were constructed, including 3 replicates at the center point a using 3-variables and 5 level CCD. Experimental errors were minimized by randomizing the experimental order in order to minimize the impact of unexplained variability. The experimental and predicted results for the DPPH radical scavenging activity (RSA), tyrosinase activity inhibition (TAI), and collagenase activity inhibition (CAI) are shown in Table 2.

To determine the correlation between the 17 experimental runs of CCD experimental conditions and the experimental results, multiple regression models were proposed to predict the optimum levels of these 3 variables. By applying multiple regression analysis to the experimental data, dependent variables (Y) and tested variables were related by the following quadratic regression equations (Table 3).

Analysis of variance (ANOVA) is a statistical test for analyzing experimental data. It subdivides the total variation in a dataset into component parts that are associated with specific sources of variation in order to test a hypothesis on the variables of the model or to estimate variance components [25]. Response surface analysis and ANOVA were employed to determine the coefficients, evaluate statistical significance of the model terms, and fit the mathematical models of the experimental data that aimed to optimize the overall region for response variables [26]. As established by the model, the correlation coefficients (R^2^) used to determine the relationship between the experimental and predicted responses by regression models were in the range of 0.8862~0.9622. This suggests that the process variables analyzed explain more than 88.6% of the independent variables. The Design Expert software was used to calculate the coefficients of the quadratic regression equations and model suitability was tested by ANOVA. According to the monomial coefficient value of quadratic regression equations are listed in Table 4 and the order of priority among the main effect of independent variables is ethanol concentration (X_3_) > extraction temperature (X_2_) > extraction time (X_1_).

### 2.2. Effect of Extraction Conditions on RSA

Table 2 shows the experimental data of RSA according to different UAE conditions. RSA of peanut shell extract was determined in the range of 7.6%~89.9%. The highest RSA was identified under the following extraction conditions: extraction time of 55.0 min, extraction temperature of 60.0 °C, and ethanol concentration of 50.0% (Run #10). The lowest RSA of 7.6%, under an extraction time of 30.0 min, extraction temperature of 60.0 °C, and ethanol concentration of 0.0%, was identified as the experimental value (Run #13). By applying multiple regression analysis, the experimental data and responses were related by quadratic regression equations (Table 3). Statistical analysis revealed that R^2^ of the regression model was 0.9308 (*p* = 0.0027), which indicates that this equation could explain 93.0% of the experimental conditions results, implying that the model was highly significant and could be used to accurately predict the response function.

**Figure 1:**
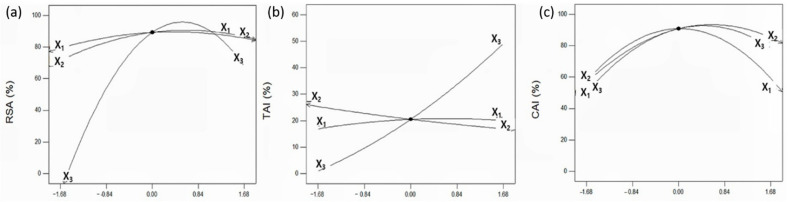
Perturbation plots for effects of extraction time (X_1_), extraction temperature (X_2_), and ethanol concentration (X_3_) on the RSA, TAI, and CAI of peanut shell extract. Perturbation plots for RSA, TAI, and CAI of peanut shell extract shows all factors at a center point by changing one factor over its range while the other factors were fixed. Perturbation plots for RSA of peanut shell extract (**a**), Perturbation plots for TAI of peanut shell extract (**b**), Perturbation plots for CAI of peanut shell extract (**c**).

**Figure 2:**
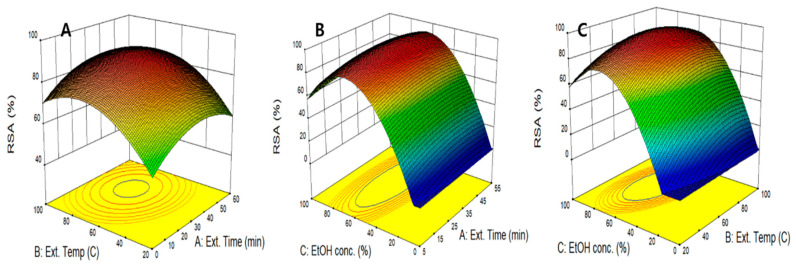
Response surface plots for RSA of peanut shell extract according to extraction time, extraction temperature, and ethanol concentration. RSA as a function of extraction temperature and extraction time (**A**), extraction time and ethanol concentration (**B**), and extraction temperature and ethanol concentration (**C**).

The effect of an individual UAE variable at fixed levels of other variables on RSA is predicted and shown in Figure 1a. RSA tends to increase and then decrease as all UAE variables increased. Ethanol concentration had the greatest effect on RSA among the three UAE variables, whereas extraction time and extraction temperature had the least effect on RSA. This result is consistent with the ANOVA results in which ethanol concentration showed a more significant effect (*p* = 0.0002) on RSA as shown in Table 4. The interaction effect between independent variables on RSA was visualized using 3D response surface curves. The extraction temperature and extraction time were changed simultaneously at the fixed level of ethanol concentration (Figure 2A). As the two variables (extraction temperature and time) increased, RSA increased to the maximum level and then decreased again. The highest RSA was obtained at an extraction temperature of 56.1 °C, which therefore suggests the extraction of bioactive compounds with antioxidant potential, such as polyphenols, increases with the destruction of plant wall component, such as lignin, at temperatures up to 56.1 °C; however, at higher temperatures, RSA was decreased due to the decomposing or polymerization of antioxidant ingredients. Figure 2B,C show that RSA was not significantly affected by the extraction time or temperature, whereas RSA was significantly affected by ethanol concentration, which was the highest at the ethanol concentration of 61.0% and which also declined again. This result is consistent with that of the hot-water extraction experiment of *Lespedeza cuneata* by Kim et al. in which RSA was more affected by ethanol concentration than extraction temperature, and RSA was the maximum at the ethanol concentration range of 60%~70% [27]. These results indicate that the extraction efficiency of the binary solvent (water and ethanol) is more effective for single solvent extraction in UAE of peanut shells.

### 2.3. Effect of Extraction Conditions on TAI

Tyrosinase is an enzyme that promotes melanin production by oxidizing tyrosine in the basement layer of the epidermis and inhibition of this enzyme is essential for the enhancement of skin-whitening [28]. The TAI of peanut shell extracted via UAE, according to 17 extraction conditions, ranged from 0.34% to 51.8% (Table 2). Based on experimental values, the relationship between independent variables (X_1_, X_2_, X_3_) and the dependent variable (TAI) was modeled using quadratic regression equations as shown in Table 3. To evaluate the agreement between the experimental and predicted values derived by the quadratic regression models, goodness-of-fit of the model was evaluated based on ANOVA. The R^2^ was 0.9622, which is close to 1 and indicates a high degree of correlation between the experimental and predicted values. *p*-value is used as a tool to evaluate the significance of each coefficient and interactions between each independent variable. The UAE variables will be more significant if the *p*-value becomes smaller and significance was confirmed at the level of *p* < 0.05 [29,30]. In evaluating the effects of independent variables, the significance was determined in the order of ethanol concentration (*p* < 0.0001) > extraction temperature (*p* < 0.0598) > extraction time (*p* < 0.4329), which confirmed that the effect of the ethanol concentration was the most significant in TAI.

**Figure 3:**
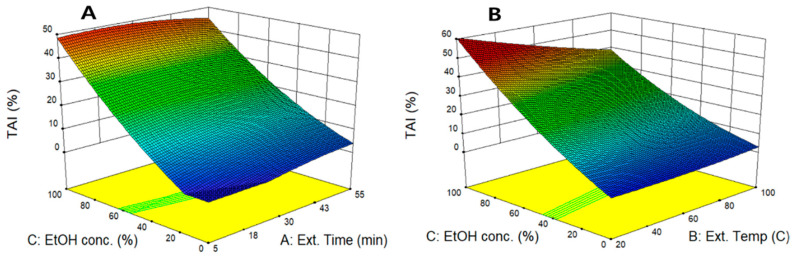
Response surface for TAI in peanut shell extract according to extraction time, extraction temperature, and ethanol concentration. TAI as a function of extraction time and ethanol concentration (**A**), extraction temperature and ethanol concentration (**B**).

To compare the effect of UAE conditions on TAI, the perturbation plot was used to evaluate the effect of individual variables on TAI by fixing two variables at center point. As shown in Figure 1b, TAI showed a different pattern compared to the previous RSA experiment; it increased as the ethanol concentration increased, while the extraction time did not significantly affect TAI. The significant proportional increase of TAI with ethanol concentration can be explained by the ANOVA results. TAI was significantly affected by the primary term of ethanol concentration (X_3_), and (*p* < 0.05) the quadratic term is not statistically significant, thus, showing a strong proportional relationship between TAI and ethanol concentration. The 3D response surface curve is the graphical representations of the quadratic regression equation and results of TAI, as affected by the extraction temperature (X_1_), extraction time (X_2_), and ethanol concentration (X_3_). Figure 3A visualizes the interaction effect of extraction time and ethanol concentration on TAI. The result confirmed that the extraction time showed no significant effect on TAI, whereas the ethanol concentration had a strong proportional relationship with TAI. Similarly, as shown in Figure 3B, TAI was more dependent on ethanol concentration than on extraction temperature and the highest TAI attained as ethanol concentration increased to 99.5%. In exploring UAE conditions for maximum TAI, the maximum TAI conditions values were predicted to be 30.0 min, 26.3 °C, and 99.5%. This result is similar to that reported by Nakamura et al. [31] In a study on biological activity of citron leaves, when 20.0%~80.0% of ethanol was used as an extraction solvent, TAI increased in proportion in response to the increase in ethanol concentration and showed maximum value in extraction using 80% ethanol. This suggests that using higher concentration of ethanol is advantageous in extracting bioactive compounds with skin-whitening effect from peanut shell or other plants.

### 2.4. Effect of Extraction Conditions on CAI

Collagen is the most abundant protein in mammals and the main structural component of the extracellular matrix with gly-pro-hyp repeating units longer than 1400 amino acids. Collagenase is an enzyme that breaks down peptide bonds of collagen that form skin, bones, tendons, and ligaments. Collagen present in dermis is decomposed by collagenase, which causes skin-wrinkles and reduces skin elasticity; therefore, it is necessary to reduce the activity of collagenase to prevent skin-wrinkles [32,33]. The optimization of the UAE condition was performed to maximize CAI of peanut shell extract. A total of 17 runs were needed for optimizing the three individual variables and the experimental data of CAI obtained under experimental sets were 25.2%~92.3% (Table 2). Based on the 17 experimental runs, by applying multiple regression analysis on the experimental data, response and independent variables were related by the following quadratic regression equation in terms of the coded parameters given in Table 3. Then, ANOVA was applied to determine the regression coefficients, statistical significance, and to fit the mathematical models. The mean-square values were calculated by dividing the sum of the squares of each variation source by their degrees of freedom, and a 95% confidence level (α = 0.05) was applied to determine the statistical significance in analysis of the quadratic model. The ANOVA results confirmed that R^2^ of the quadratic regression equation was 0.8862 and that the *p* value was 0.0134, which is less than the significance level (*p* < 0.05), thus indicating a good model of fit and statistical significance for predicting CAI values. In the primary term, the X_2_ and X_3_ showed significant effects and the interaction effect terms were significant in the X_1_X_2_ and X_2_X_3_ (*p* < 0.05). The effect of UAE conditions on CAI production was confirmed to be in the order of: extraction temperature (*p* = 0.0236) > ethanol concentration (*p* = 0.0240) > extraction time (*p* = 0.8505), thus indicating that the effect of extraction temperature and ethanol concentration were significant on CAI.

**Figure 4:**
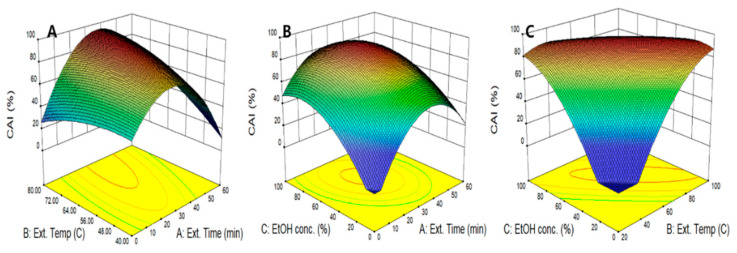
Response surface plots for CAI of peanut shell extracts according to extraction time, extraction temperature, and ethanol concentration. CAI as a function of extraction temperature and extraction time (**A**), extraction time and ethanol concentration (**B**), and extraction temperature and ethanol concentration (**C**).

Figure 1c shows a perturbation plot in which two variables are fixed and it visualized the effect of a single variable on CAI. The effects of all three variables on CAI were shown to be similar, and the three variables showed significant effects and increased and subsequently decreased CAI as each independent variable increased. In our study, 3D surface response curves were developed to visualize the interaction of two independent variables in CAI using quadratic regression equations (Figure 4). When the ethanol concentration was fixed at the center point, the effect of extraction time and temperature on CAI was evaluated in Figure 4A. As the two variables changed simultaneously, CAI increased to 33.4 min and 76.8 °C and decreased again after a maximum CAI of 92.8%. As shown in Figure 4B,C, CAI had the highest value at the ethanol concentration of 64.3%, showing a gradual decreasing tendency afterward, which suggests that a binary solvent consisting 64.3% of ethanol is more suitable as an extraction solvent. This result is consistent with previous research that reported that a binary solvent of water and ethanol showed a higher CAI than water in the extraction of bioactive compounds from *Orostachys japonica*, which suggests that 50% ethanol would be more advantageous in extracting skin-whitening ingredients [34]. The maximum CAI of the peanut shell extract predicted by the quadratic regression model was 94.5%, which was obtained under conditions of extraction time of 45.1 min, extraction temperature of 93.6 °C, and ethanol concentration of 42.3%. The CAI obtained in our study was 94.5%, which is more than twice the effects of 39.4% and 40.3% of CAI values of green tea extracts reported by Oh et al. [35].

### 2.5. Optimum Extraction Conditions

**Figure 5:**
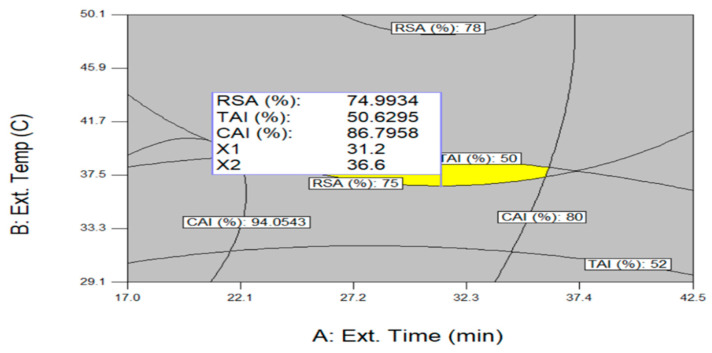
Superimposing contour map for simultaneous optimization of three variables for the maximization of RSA (%), TAI (%), and CAI (%). Ethanol concentration was fixed at the optimum level of 93.2%.

Antioxidant, skin-whitening, and anti-wrinkle effects are all important functions for cosmetics and it is necessary to derive conditions that can maximize these three functions simultaneously in optimizing UAE conditions. Figure 5 shows an optimization procedure that can simultaneously maximize RSA (Y_1_), TAI (Y_2_), and CAI (Y_3_) by overlapping each optimal condition of a contour graph derived through a quadratic regression equation. The ranges of independent variables for the optimization of three variables were limited to the extraction time of 5.0~55.0 min, extraction temperature of 26.0~94.0 °C, and ethanol concentration of 0.0%~99.5% (Table 5). According to individual optimal extraction conditions, optimum UAE conditions were 31.2 min of extraction time, 36.6 °C of extraction temperature, 93.2% of ethanol concentration and, under the above conditions, RSA of 74.9%, TAI of 50.6%, and CAI of 86.8% were predicted. When the predicted RSA, TAI, and CAI values were compared to those obtained from the experiment for validation, the values from the validation test were similar to those of the predicted values, where the values were 78.2%, 52.3%, and 87.7%, respectively.

### 2.6. Comparison of SE and UAE

To confirm the extraction effectiveness of UAE, we compare RSA, TAI, and CAI of peanut shell extract produced using UAE and Soxhlet extraction (SE) techniques. When the SE was conducted under general SE conditions using 99.5% ethanol at 70 °C for 4 h of extraction time, RSA, TAI, and CAI were found to be 75.5%, 60.2%, and 74.4%, which were not much different from the results obtained under optimal UAE condition. However, when the SE conditions were set equal to the UAE optimal conditions of 31.2 min and 93.2% ethanol, RSA, TAI, and CAI decreased by 62.0, 28.3, and 45.6%, respectively, compared to UAE under optimal condition. The advantage of ultrasound in producing useful materials from peanut shells was evaluated as a process suitable for high productivity and industrialization due to low solvent consumption and short extraction time.

### 2.7. mRNA Expression of MMP-3 and TRP-1

**Figure 6:**
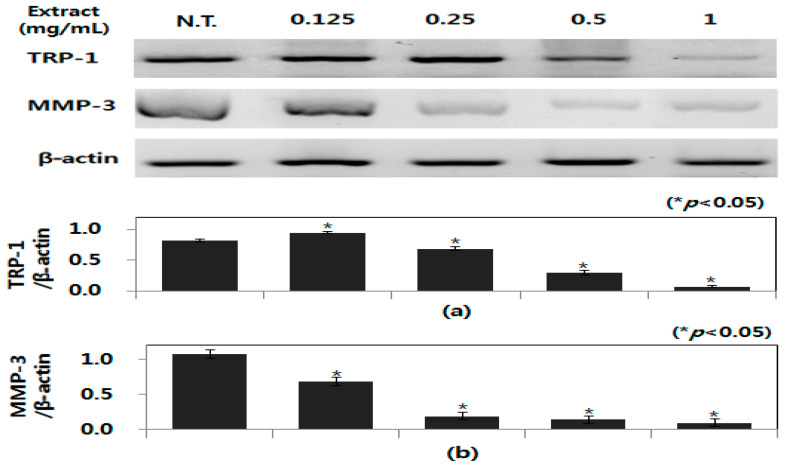
Effect of peanut shell extract on the expression of TRP-1 and MMP-3 mRNA. B16-F0 cells were treated with various concentrations of peanut shell extract for 24 h. The mRNA levels of MMP-9 and TRP-1 were measured using RT-PCR (**a**). Band intensities were estimated using Quantity One software and normalized to β-actin (* *p* < 0.05). All data are expressed as mean ± SD of three separate experiments performed in triplicate. N.T = not treated sample, Quantification of TRP-1 mRNA expression (**a**), quantification of MMP-3 mRNA expression (**b**).

In mammalian melanocytes, melanogenesis and collagen hydrolysis are controlled by TRP and MMP genes, respectively, and TRP-1 and MMP-3 are known as the main genes for the regulation of melanogenesis and collagen hydrolysis; therefore, RT-PCR analysis on whole-cell lysates of B16-F0 cells was performed and the effect of peanut shell extract produced from UAE under optimal conditions (31.2 min, 36.6 °C, 93.2%) on mRNA expression of MMP-3 and TRP-1 was studied. As Figure 6 shows, peanut shell extract significantly downregulated the expression of MMP-3 and TRP-1 in B16-F0 cells when the gene expression experiments were performed with a peanut shell extract concentration range of 0~1 mg/mL. The peanut shell extract significantly reduced MMP-3 and TRP-1 expression by 6.1-fold and 8.7-fold, respectively, at 1.0 mg/mL. These results suggest that peanut shell extract inhibits collagen degradation in B16F0 cells by inactivation of MMP-3 to inactivation of MMP-1 and interfere with cooperation of MMP-9 [36]. Existing studies have shown that treatment with plant extracts inhibited the expression of microphthalmia-associated transcription factor (MITF) by phosphorylating extracellular signal-regulated protein kinase (ERK). Thus, the inhibitory effect of melanin production by peanut shell extract is attributed to the inhibition of tyrosinase activity through expression inhibition of ERK and MITF [37]. Thus, peanut shell extracts reduced mRNA expression levels of TRP-1 and MMP-3, which indicates that peanut shell extract possesses strong inhibitory activities on collagenolysis and melanogenesis making it an excellent cosmetic material with skin-whitening and anti-wrinkle effects.

## 3. Materials and Methods

### 3.1. Materials and Reagents

Peanut shells were purchased from Nonghyup mart (Gochang, Jeonbuk, Korea) in March 2019 and the shells were dried at 60 °C using dry oven (FC 49, Lab House, Seoul, Korea) for 24 h until the dry weight remained constant. Dried peanut shells were pulverized using a food processor (Hanil HMF-3800, Seoul, Korea) and then passed through a 600 μm sieve. Ethanol was purchased from Samchun chemical (95.0% *v/v*, Seoul, Korea). Folin–Ciocalteu reagent, gallic acid (97%), and quercetin were purchased from Merck (Kenilworth, NJ, USA). 2,2-Diphenyl-1-picrylhydrazyl (DPPH), ascorbic acid, and 3,4-dihydroxy-l-phenylalanine (L-DOPA) were purchased from Sigma-Aldrich (St. Louis, MO, USA). All other chemicals used in this experiment were of analytical grade and purchased from Sigma-Aldrich. All the stock solutions were prepared by purified deionized water using a Milli-Q purification system (Millipore, Burlington, VT, USA).

### 3.2. Ultrasound-Assisted Extraction and Soxhlet Extraction

Powdered peanut shell (1 g) was placed into an extraction vessel, each with 10 mL of solvent and mixed using vortex mixer (VM-10, Daihan Scientific Co., Ltd., Wonju, Korea) for 1 min. Extraction was carried out by circulating water in the ultrasonic extractor (250 W, SD-D250H, Daihan Scientific Co., Ltd., Wonju, Korea) using an external refrigerated bath circulator (CDRC8, Daihan Scientific Co., Ltd., Wonju, Korea) with a digital timer and a temperature controller. The extraction was performed with the ultrasonic device equipped with a digital timer and a temperature controller. Sample were sonicated for various experimental durations and temperatures at working frequency of 40 kHz. Then, the extract was centrifuged at 10,000 rpm for 10 min (236R, Labogene, Seoul, Korea). After centrifugation, the sample volumes were made up to 5 mL and filtered through a 0.2 μm membrane filter prior to analysis. For the Soxhlet extraction, the powdered peanut shell (5 g) were continuously extracted with 100 mL using 99.5% ethanol for 4 h (8 cycles) at a maximum temperature of 70 °C in a Soxhlet apparatus. The ultrasound-assisted extraction technique was shown to be very efficient in the extraction of oil from grape seeds the advantage of the ultrasound, compared to the conventional extraction methods both for oil and polyphenols, was similar since oil/polyphenols yield obtained with a lower solvent consumption and a shorter extraction time.

### 3.3. Experimental Design

The experimental design was carried out using CCD, a type of RSM to minimize the number of experimental runs and study the interaction between the factors. The Design-Expert^®^ software 8.0 (State-Ease, City, MN, USA) was used for the design of experiments, data analysis, and optimization of extraction conditions for the maximization of the extraction of bioactive compounds having antioxidant, skin-whitening, and anti-wrinkle effects from peanut shell. The experiments were designed according to CCD, the range and center point values of three independent variables presented were based on the results of preliminary experiments (Table 1). The CCD was applied to predict the optimal UAE conditions for the maximization of responses including RSA, TAI, and CAI from peanut shells. As independent variables, the three variables chosen were extraction time (X_1_), extraction temperature (X_2_), and ethanol concentration (X_3_). A total of 17 experimental runs were generated with three replications at the central points to estimate the reproducibility. The quadratic regression model was used to fit the experimental data and applied to predict the response variables, as shown in Equation (1):

*Y* = β_0_ + β_1_X_1_ + β_2_X_2_ + β_3_X_3_ + β_11_X_12_ + β_22_X_22_ + β_33_X_32_ + β_12_X_1_X_2_ + β_13_X_1_X_3_ + β_23_X_2_X_3_ (1)

where *Y* is the predicted response; β_0_ is the constant (intercept); β_1_, β_2_, and β_3_ are the regression coefficients for the linear effect terms; β_11_, β_22_, and β_33_ are the quadratic effect terms; and β_12_, β_13_, and β_23_ are the interaction effect terms, respectively. A response surface analysis and the ANOVA were employed to determine the regression coefficients and statistical significance of the model terms and to fit the mathematical models of the experimental [38].

### 3.4. DPPH Radical Scavenging Activity (RSA)

RSA of the peanut shell extract was as described by Pereira-Caro et al. [39]. Solution of 0.01 mM DPPH in methanol (95%) was prepared and 1.25 mL was added to 0.25 mL of diluted extract. RSA was determined measuring absorbance at 517 nm using UV-Vis spectrophotometer (UV1650PC, Shimadzu, Kyoto, Japan) after 20 min of incubation. Blank was prepared using distilled water and the RSA was calculated according to the below. (Equation (2)):

(2) $$\mathrm{RSA}\;\left(\%\right)=\left\{1-\frac{\mathrm{Abs}\;\left(\mathrm{sample}\right)}{\mathrm{Abs}\;\left(\mathrm{control}\right)}\right\}\times 100$$

### 3.5. Tyrosinase Activity Inhibition (TAI)

TAI was performed according to the modified method using L-DOPA as substrate by Jo et al. [40]. Samples were mixed with 200 µL L-DOPA and 200 µL potassium phosphate buffer (pH 6.8) and 200 µL of tyrosinase (125 U/mL) was added in the test tube and incubated at 37 °C for 20 min. The sample absorbance was measured at 475 nm using a UV-Vis spectrophotometer and the results were compared to the control. For each concentration, enzyme activity was calculated as a percentage compared to that of the assay using buffer without any inhibitor and TAI was calculated based on the following formula. (Equation (3)):

(3) $$\mathrm{TAI}\;\left(\%\right)=\left\{1-\frac{\mathrm{Abs}\;\left(\mathrm{control}\right)-\mathrm{Abs}\;\left(\mathrm{sample}\right)}{\mathrm{Abs}\;\left(\mathrm{control}\right)}\right\}\times 100$$

where Abs (control) is the absorbance of buffer + collagenase; Abs (sample) is the absorbance of buffer + collagenase + sample/standard.

### 3.6. Collagenase Activity Inhibition (CAI)

The measurement of CAI of extracts was carried out by modifying the methods of Wünsch and Heindrich [41]. The substrate, 4-phenylazobezyloxylcarbonyl-Pro-Leu-Gly-Pro-Arg (FALGPA), was dissolved in 10 mL buffer to 1.2 mg/mL and then 125 μL of solution was added and incubated for 60 min at 37 °C. Collagenase was dissolved in the buffer to 0.4 mg/mL, and 75 μL of enzyme solution was added to buffer solution. The enzyme-substrate mixture was incubated in a water bath at 37 °C for 30 min and the reaction was stopped by adding 75 μL of 20% citric acid (*w*/*v*). After adding 1.5 mL of ethyl acetate, the ethyl acetate layer was separated and absorbance was measured at 320 nm. The percent of inhibition was calculated according to the following formula.

(4) $$\mathrm{CAI}\;\left(\%\right)=\left\{1-\frac{\mathrm{Abs}\;\left(\mathrm{control}\right)-\mathrm{Abs}\;\left(\mathrm{sample}\right)}{\mathrm{Abs}\;\left(\mathrm{control}\right)}\right\}\times 100$$

where Abs (control) is the absorbance of buffer + collagenase; Abs (sample) is the absorbance of buffer + collagenase + sample/standard.

### 3.7. Maintenance and Culturing of Cell Lines

Melanin-producing B16-F0 melanoma cell was obtained from Korea Cell Line Bank (KCLB, Chongno, Seoul, Korea) and was cultured in Dulbecco’s modified Eagle’s medium (DMEM, Sigma-Aldrich, St. Louis, MO, USA) supplemented with fetal bovine serum (FBS, 10%, Welgene, Gyeongsan, Korea) and antibiotic solution of penicillin-streptomycin (Sigma-Aldrich, St. Louis, MO, USA). Trypsin-EDTA (Gibco, Grand Island, NY, USA) was used for trypsinization of cells. All materials used were of cell culture grade.

### 3.8. Reverse Transcription Polymerase Chain Reaction (RT-PCR)

RT-PCR was performed to measure changes in MMP-3 and TRP-1 gene expression levels associated with whitening and anti-wrinkle effects, B16-F0 cells were cultured in a 24-well plate treated with the different concentrations of peanut shell extract in serum-free DMEM, and incubated for 24 h. The untreated cell control was maintained under the same conditions as the tested group during the experiment. RNA isolation from cells was conducted using the AccuPrep^®^ Universal RNA Extraction Kit (Bioneer, Daejeon, Korea). Complementary DNA was synthesized using AmfiRiert Platinum cDNA synthesis Master Mix (GenDEPOT, Barker, TX, USA). RT-PCR analysis was performed using the CFX 96 touch PCR System (Bio-Rad, Hercules, CA, USA) to determine mRNA levels. The primers used were as follows: MMP-3 sense, 5′-AGTTTGGTGTCGCGGAGCAC-3′ and antisense, 5′-TACATGAGCGCTTCCGGCAC-3′; and TRP-1 sense, 5′-GCTGCAGGAGCCTTCTTTCTC-3′ and antisense, 5′-AAGACGCTGCACTGCTGGTCT-3′. An appropriate set of primers mentioned above were used to amplify respective genes using the following cycling conditions: 94 °C for 5 min, followed by 25 cycles at 95 °C for 5 s, 60 °C for 30 s (for MMP-3) and 60 °C for 30 s (for TRP-1), and 72 °C for 30 s extension. The PCR products were electrophoresed on a 1% agarose gel, stained with ethidium bromide, and visualized by using Gel Doc TM XR+ System and Quantity One software 2.0 (Bio-Rad, Hercules, CA, USA). A housekeeping protein, β-actin, was used as a loading control with the assumption that the expression levels of these proteins remains constant.

## 4. Conclusions

In this study, the complementary approach was employed for recovery and uses of bioactive substances from agricultural byproducts of peanut shell to develop added-value ingredients with multiple uses. First of all, we attempted to increase the extraction efficiency of bioactive compounds with antioxidant, skin-whitening, and anti-wrinkle effects by optimizing the UAE process. Therefore, this study employed UAE for efficient production of bioactive compounds with skin-whitening and anti-wrinkle effects from peanut shell and applied statistically-based optimization to maximize RSA, TAI, and CAI simultaneously. The UAE conditions were optimized using CCD and it was confirmed that the choice of solvent and concentration should be considered in extraction of bioactive compounds from peanut shells. By overlapping the response surfaces, curves of three dependent variables, an extraction time of 31.2 min, extraction temperature of 36.6 °C, and ethanol concentration of 93.2% were determined to be the optimal conditions of UAE. It has been confirmed that RSA of peanut shell extracts is very high and can be expected to increase in TAI and CAI, which are indicators of skin-whitening and anti-wrinkle effects, respectively. The optimization of UAE conditions confirmed an increase in the production of bioactive substances in peanut shells, and whitening and anti-wrinkle activities of peanut shell extracts through tyrosinase and collagenase activity downregulations. Based on this, the effect of peanut shells on the expression levels of MMP and TRP were evaluated to assess whether they have whitening and anti-wrinkle effects at the gene expression level. Whitening and anti-wrinkle effects of peanut shell extracts were confirmed through the downregulation of mRNA expressions as well as the inhibition of protein expressions of MMP-3 and TRP-1. Therefore, peanut shell extract has been shown to be effective in whitening and wrinkle improvement at protein expression and gene levels. Peanut shell extract, using UAE, has high antioxidant activity and excellent skin-whitening and anti-wrinkle effects, giving peanut shell great potential as a natural cosmetic and food ingredient. Furthermore, it is believed that the production of bioactive compounds using UAE can be applied to the commercialization process for the production of cosmetics, food, and pharmaceutical materials, given the higher production yield and reduced processing costs compared to conventional processes.

## Figures and Tables

**Table 1:** The central composite design (CCD) for optimization of ultrasound-assisted extraction (UAE) conditions of peanut shell.

X_i_	Independent Variable	Coded Levels				
		−1.68	−1	0	+1	+1.68
X_1_	Extraction time (min)	5.0	15.0	30.0	45.0	55.0
X_2_	Extraction temperature (°C)	26.0	40.0	60.0	80.0	94.0
X_3_	Ethanol concentration (% *v*/*v*)	0.0	20.0	50.0	80.0	99.5

Level of each variable was established based on preliminary experiments based on one-factor-at-a-time method. The distance of the axial points from the center point was ±1.68.

**Table 2:** Experimental and predicted data on radical scavenging activity (RSA), TAI, and CAI of peanut shell extract by CCD.

Run No.	Extraction Conditions			Experimental Values			Predicted Values		
	X_1_	X_2_	X_3_	RSA (%)	TAI (%)	CAI (%)	RSA (%)	TAI (%)	CAI (%)
1	15.0	40.0	20.0	14.4	7.40	25.2	26.3	5.47	35.3
2	45.0	40.0	20.0	20.7	10.6	33.8	32.3	10.5	27.6
3	15.0	80.0	20.0	33.4	6.43	68.4	43.2	4.87	62.5
4	45.0	80.0	20.0	42.1	4.56	84.1	49.0	8.29	92.0
5	15.0	40.0	80.0	82.0	42.3	91.4	83.9	40.0	82.4
6	45.0	40.0	80.0	86.5	37.3	50.4	85.6	40.3	63.6
7	15.0	80.0	80.0	87.7	30.8	46.9	79.3	32.3	60.4
8	45.0	80.0	80.0	89.7	27.6	73.2	86.6	30.1	70.5
9	5.0	60.0	50.0	87.5	13.7	64.0	79.3	16.9	57.3
10	55.0	60.0	50.0	89.9	25.3	62.9	85.5	20.0	59.1
11	30.0	26.0	50.0	81.9	23.9	62.7	71.5	25.3	56.4
12	30.0	94.0	50.0	88.8	20.5	89.3	87.7	17.0	85.1
13	30.0	60.0	0.0	7.55	0.34	50.1	12.1	0.95	50.0
14	30.0	60.0	99.5	60.8	51.8	89.0	67.9	49.1	78.6
15	30.0	60.0	50.0	88.7	20.8	90.5	89.2	20.4	90.7
16	30.0	60.0	50.0	88.6	18.2	92.3	89.2	20.4	90.7
17	30.0	60.0	50.0	88.2	22.1	87.7	89.2	20.4	90.7

No.: randomly selected experimental number; X_1_: Extraction time; X_2_: Extraction temperature; X_3_: Ethanol concentration; RSA (DPPH radical scavenging activity); TAI (tyrosinase activity inhibition); CAI (collagenase activity inhibition).

**Table 3:** Polynomial regression equations calculated by CCD for the optimization of UAE conditions of peanut shell.

Responses	Second-Order Regression Models	*R^2^	P
RSA (%)	*Y*_RSA_ = −93.85411 + 0.88251X_1_ + 1.62214X_2_ + 3.70693X_3_ − 0.010569X_1_^2^ − 5.07511X_1_X_2_ − 8.90542X_2_^2^ − 2.40222X_1_X_3_ − 6.57227X_2_X_3_ − 0.024412X_3_^2^	0.9308	0.0027
TAI (%)	*Y*_T__A__I_ = −9.34253 + 0.46766X_1_ − 9.54387X_2_ + 0.55800X_3_ − 3.19295X_1_^2^ − 1.35612X_1_X_2_ + 6.13252X_2_^2^ − 2.66703X_1_X_3_ − 2.94955X_2_X_3_ + 1.76988X_3_^2^	0.9622	0.0004
CAI (%)	*Y*_C__A__I_ = −118.02907 + 1.78059X_1_ + 2.80277X_2_ + 3.09895X_3_ − 0.051048X_1_^2^ + 0.031083X_1_X_2_ − 0.017564X_2_^2^ − 0.010883X_1_X_3_ − 0.024017X_2_X_3_ − 0.010465X_3_^2^	0.8862	0.0134

*R^2^ (coefficient of determination), P (probability value of model); Y is the predicted response.

**Table 4:** ANOVA of the experimental results of CCD for full quadratic models.

	RSA			TAI			CAI		
	Sum of Squares	F Value	*p* Value	Sum of Squares	F Value	*p* Value	Sum of Squares	F Value	*p* Value
Model	13,775.91	10.46	0.0027	2982.57	19.82	0.0004	6525.54	6.06	0.0134
X_1_	48.20	0.33	0.5839	11.57	0.69	0.4329	4.58	0.038	0.8505
X_2_	272.52	1.86	0.2145	84.15	5.03	0.0598	994.50	8.31	0.0236
X_3_	7823.52	53.49	0.0002	2797.79	167.37	<0.0001	993.96	8.31	0.0240
X_1_X_2_	0.0018	<0.0001	0.9973	1.32	0.079	0.7865	695.64	5.81	0.0467
X_1_X_3_	9.35	0.064	0.8077	11.52	0.69	0.4338	191.86	1.60	0.2459
X_2_X_3_	124.4	0.85	0.3871	25.06	1.50	0.2604	1661.26	13.88	0.0074
X_1_^2^	63.82	0.44	0.5300	5.83	0.35	0.5735	1488.97	12.44	0.0096
X_2_^2^	143.22	0.98	0.3554	0.68	0.041	0.8460	557.13	4.66	0.0678
X_3_^2^	5366.62	36.69	0.0005	28.21	1.69	0.2351	986.28	8.24	0.0240

X_1_: Extraction time; X_2_: Extraction temperature; X_3_: Ethanol concentration; Y_1_: RSA; Y_2_: TAI; Y_3_: CAI.

**Table 5:** Comparison of RSA, TAI, and CAI of peanut shell extract obtained by Soxhlet extraction (SE) and ultrasound-assisted extraction (UAE) at different extraction conditions.

	UAE 1 ^1^	UAE 2 ^2^	SE 1	SE 2
Extraction time (min)	30.0	31.2	31.2	240.0
Extraction temperature (°C)	60.0	36.6	70.0	70.0
Ethanol concentration (%)	50.0	93.2	93.2	99.5
RSA (%)	88.5 ± 0.26	78.2 ± 1.96	48.5 ± 1.35	75.5 ± 1.43
TAI (%)	20.4 ± 1.99	52.3 ± 2.30	14.8 ± 0.27	60.2 ± 2.71
CAI (%)	92.3 ± 1.32	87.7 ± 2.59	40.0 ± 1.02	74.4 ± 2.10
Total	201.2	218.2	103.3	210.1

^1^ Ultrasound-assisted extraction of peanut shell under center point of CCD (Run No. 15, 16, 17); ^2^ ultrasound-assisted extraction of peanut shell under optimal extraction condition.

## Data Availability

No new data were created or analyzed in this study. Data sharing is not applicable to this article.
